# An Innovative 3D Printed Tooth Reduction Guide for Precise Dental Ceramic Veneers

**DOI:** 10.3390/jfb14040216

**Published:** 2023-04-12

**Authors:** Manuel Robles, Carlos A. Jurado, Francisco X. Azpiazu-Flores, Jose Villalobos-Tinoco, Kelvin I. Afrashtehfar, Nicholas G. Fischer

**Affiliations:** 1 Department of Restorative Dentistry, Facultad de Odontologia, Universidad Vizcaya de las Americas, Hermosillo 83240, Mexico; 2 Department of Prosthodontics, The University of Iowa College of Dentistry and Dental Clinics, Iowa City, IA 52242, USA; 3 Department of Restorative Dentistry, Dr. Gerald Niznick College of Dentistry, University of Manitoba, Winnipeg, MB R3E 0W3, Canada; 4 Postgraduate Program in Periodontology and Implant Surgery, Facultad de Odontologia, Universidad Nacional de Rosario, Rosario S2002KTT, Argentina; 5 Evidence-Based Practice Unit, Clinical Sciences Department, College of Dentistry, Ajman University, Ajman City P.O. Box 346, United Arab Emirates; 6 Department of Reconstructive Dentistry and Gerodontology, School of Dental Medicine, University of Bern, 3010 Bern, Switzerland; 7 Minnesota Dental Research Center for Biomaterials and Biomechanics, University of Minnesota School of Dentistry, Minneapolis, MN 55108, USA

**Keywords:** permanent dental restoration, dental veneers, dental prosthesis design, computer-aided design, three-dimensional printing, prosthodontic tooth preparation, dental porcelain, dental bonding, dental materials, dental esthetics, CAD-CAM

## Abstract

Tooth reduction guides allow clinicians to obtain the ideal space required for ceramic restorations. This case report describes a novel design (CAD) for an additive computer-aided manufactured (a-CAM) tooth reduction guide with channels that permitted access for the preparation and evaluation of the reduction with the same guide. The guide features innovative vertical and horizontal channels that permit comprehensive access for preparation and evaluation of the reduction with a periodontal probe, ensuring uniform tooth reduction and avoiding overpreparation. This approach was successfully applied to a female patient with non-carious lesions and white spot lesions, resulting in minimally invasive tooth preparations and hand-crafted laminate veneer restorations that met the patient’s aesthetic demands while preserving tooth structure. Compared to traditional silicone reduction guides, this novel design offers greater flexibility, enabling clinicians to evaluate tooth reduction in all directions and providing a more comprehensive assessment. Overall, this 3D printed tooth reduction guide represents a significant advancement in dental restoration technology, offering clinicians a useful tool for achieving optimal outcomes with minimal tooth reduction. Future work is warranted to compare tooth reductions and preparation time for this guide to other 3D printed guides.

## 1. Introduction

The smile is a crucial aspect of facial aesthetics, and many studies have demonstrated that an attractive smile can have a positive impact on a person’s well-being [1,2,3,4]. With the rise of social media, the desire for ideal smiles has increased, leading to the widespread adoption of elective cosmetic dental treatments [5,6]. As a result, modern dentistry has expanded beyond treating oral diseases, emergencies, and restoring damaged dentition [7,8]. Among elective treatments, laminate veneers have become a highly demanded option [9,10,11]. However, it is recommended that clinicians take a conservative approach to elective dental procedures, allowing for possible retreatment if necessary [12,13].

Clinicians can perform veneer tooth preparations with different techniques such as freehand, using depth cut/groves, or sectioned tooth reduction guides. The use of depth grooves is a technique in which clinician makes longitudinal [14] or horizontal [15] depth orientation grooves with a small round bur or with specially designed depth gauge burs [16,17,18]. Tooth reduction guides are another approach in which a guide is fabricated with polyvinylsiloxane (PVS) putty material [19,20], resin composite [21], metal [22], or a thermoplastic polymer [23]. This approach involves measuring the amount of tooth reduction and its uniformity, thus ensuring prostheses that provide optimum periodontal health, aesthetics, and structural durability [24,25,26]. Lastly, freehand techniques may be employed by experienced clinicians capable of visualizing the amount of tooth removal and space needed for the ceramic veneer without any reference. A recent study evaluated the free-hand, silicone guide, thermoplastic guide, and two different types of 3D printed tooth reduction guides and concluded that any type of tooth reduction guide provided more accuracy for veneer preparation than freehand preparation [27]. Therefore, it can be suggested that tooth reduction should be performed with a reduction aid in order to guarantee a controlled tooth reduction.

Additive manufacturing allows converting a digital 3D design into a physical object through a layer-by-layer process. In dentistry, three-dimensional (3D) printing offers a more efficient fabrication workflow than traditional techniques and permits less labor-intensive and time-saving manufacturing procedures [28]. A recent study evaluated the accuracy of conventional casts fabricated from light and heavy-body polyvinyl siloxane material and 3D printed casts from five different printers. Trueness and precision were analyzed with 3D analysis software. The results concluded that the 3D printed models were more precise than the models obtained from conventional elastomeric impressions [29]. Currently, contemporary digital technology allows the clinician to design precise tooth reduction guides digitally and manufacture them using a photopolymer resin within minutes [30]. The literature shows that 3D printed tooth reduction guides can be used successfully to evaluate specific surfaces such as incisal, facial, and interproximal; however, no guide has been designed to evaluate multiple surfaces at the same time. The aim of this case report is to describe a transparent tooth reduction guide with horizontal, vertical, and incisal channels which permitted evaluating the tooth preparation for veneers more objectively since a clear visualization of the teeth contours and the projected restorations was possible.

## 2. Case Report

**Figure 1:**
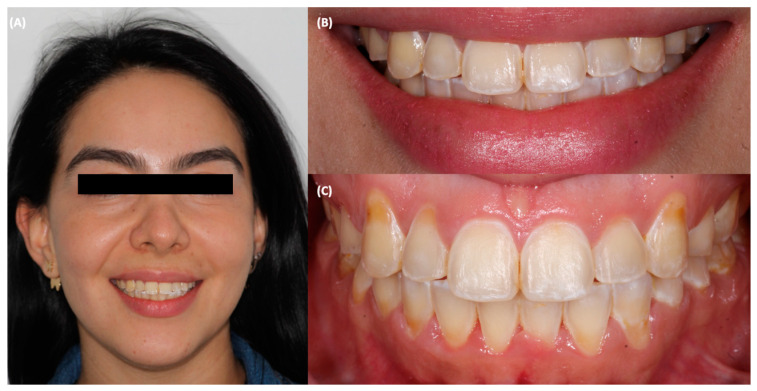
Initial situation. (**A**) face smiling, (**B**) smile, and (**C**) intraoral image.

A 31-year-old female patient went to the clinic and was interested in improving the symmetry and esthetics of her smile (Figure 1).

**Figure 2:**
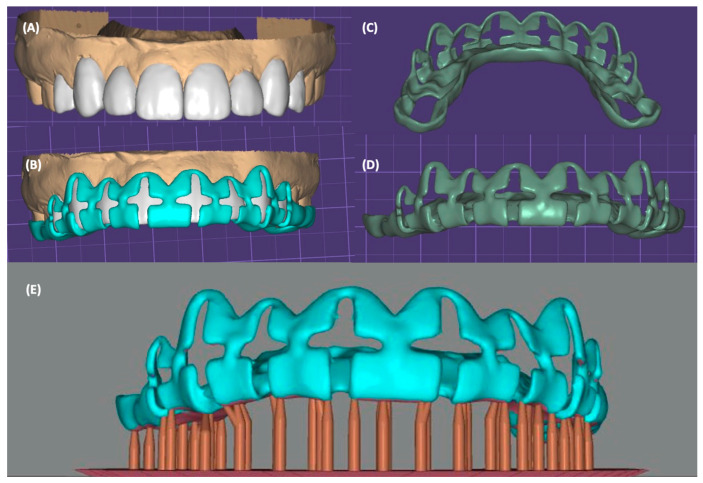
Digital wax-up and reduction guide. (**A**) Additive digital wax-up, (**B**) digital tooth reduction guide designed over wax-up, (**C**) lingual view of reduction guide, (**D**) facial view of reduction guide, and (**E**) reduction guide ready for printing.

After clinical evaluation, the patient was diagnosed with non-carious cervical lesions on the maxillary right canine, right lateral incisor, and left canine. Additionally, white spots were noted on the incisal edges of all anterior teeth. Gingival recession was also noted on the maxillary canines and right lateral incisor. Extraorally, a low smile without gingival display was determined. The option of performing soft-tissue grafts for both canines and maxillary right lateral incisor followed by ceramic veneers from canine to canine was presented to the patient. The patient declined surgical procedures and requested only ceramic restorations. The low lip smile enabled “hiding” the asymmetrical gingival zeniths and not compromise the final esthetic outcome. Diagnostic impressions were made with polyvinylsiloxane impression material (Virtual, Ivoclar Vivadent, Schaan, Liechtenstein) and were poured up with type IV stone (Fujirock, GC Corporation, Tokyo, Japan). The maxillary and mandibular casts were scanned with a laboratory 3D scanner (Degree of Freedom HD, DOF, Seoul, Republic of Korea), and digital teeth arrangements for the anterior teeth were made in professional dental design software (Exocad 2.4, Exocad GmbH, Darmstadt, Germany). In the same software, the digital additive wax-up and the tooth reduction guide were designed. The design included vertical and horizontal channels with a width of 3 mm and depth of 1 mm; the vertical access was extended to the proposed incisal edge with a 1.5 mm opening (Figure 2).

**Figure 3:**
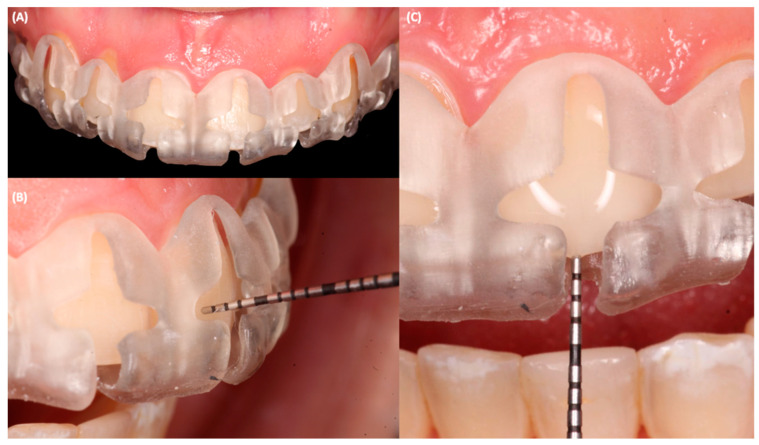
Printed tooth reduction guide in the mouth. (**A**) Frontal view, (**B**) lateral view with periodontal probe measuring, and (**C**) frontal view of the measurement on the incisal edge during tooth preparation.

The tooth reduction guide was printed in a liquid crystal display (LCD) 3D printer (Anycubic Resin 3D Printer Mono 4K, Anycubic, Shenzhen, China) with transparent photopolymerizable resin (Anycubic Clear UV Resin, Anycubic, Shenzhen, China). Subsequently, a diagnostic mock-up was placed with temporary bis-acrylic material (Structure Premium, VOCO, Cuxhaven, Germany) and conservative tooth preparations were performed first with fine diamond bur (801 Spherical, JOTA AG, Rüthi, Switzerland) following the vertical and horizontal depth grooves with a 0.5 mm reduction, in addition to a 1.5 mm reduction for the incisal area. Then, the tooth reduction guide was removed, and the remaining portions of the tooth were prepared. During this process, the guide was placed on and off to re-verify the amount of tooth structure removed (Figure 3).

**Figure 4:**
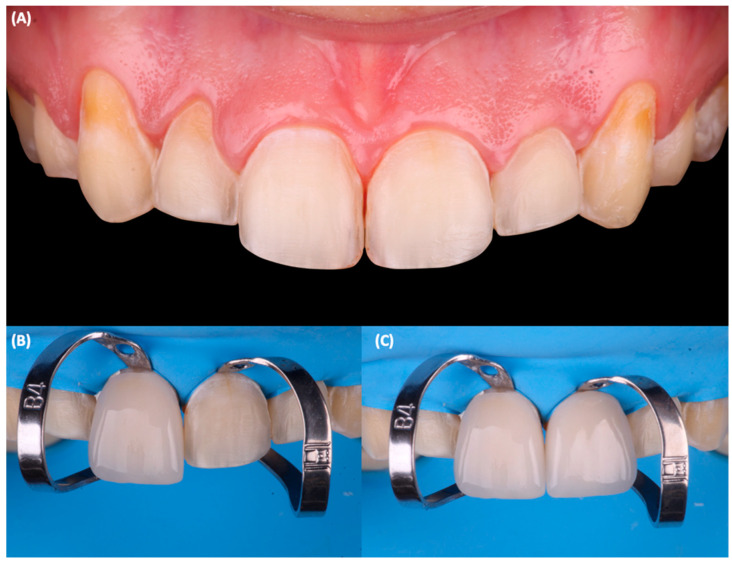
Final tooth preparations and bonding of the restorations. (**A**) Final frontal view, (**B**) cementation for the right central incisor veneer, (**C**) cementation for the left central incisor veneer.

**Figure 5:**
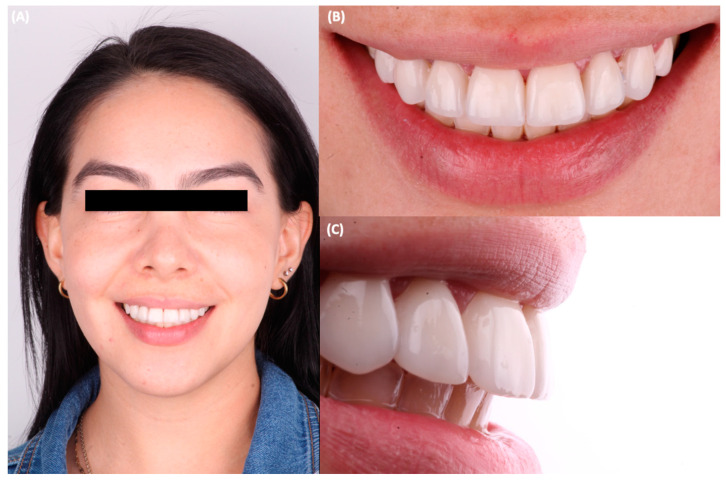
Final result. (**A**) Face smiling, (**B**) smile frontal view, and (**C**) smile right side view.

**Figure 6:**
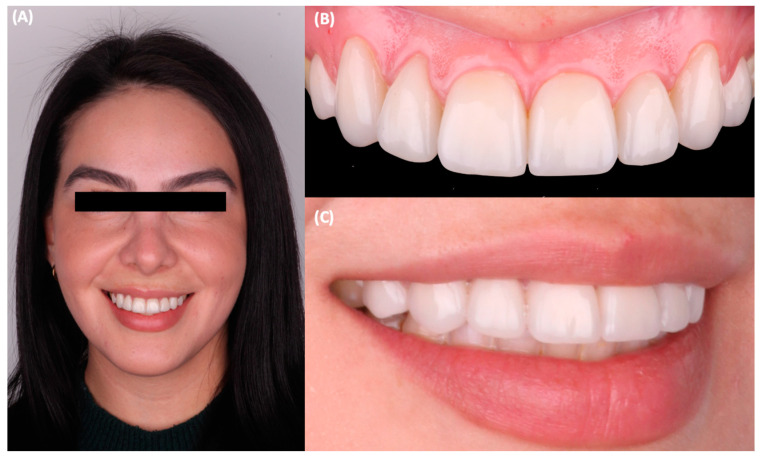
Follow-up at one year. (**A**) Face smiling, (**B**) intra-oral frontal view, and (**C**) smile right side view.

Final tooth preparations were polished with coarse, medium, and fine polishing discs (Sof-Lex XT Disc, 3M Oral Care, St Paul, MN, USA), and the final impressions were made with silicone putty (Hydrorise Putty, Zhermack, Badia Polesine, Italy) and light body PVS (Hydrorise Putty, Zhermack, Badia Polesine, Italy). The master cast was fabricated out of type IV stone (Fujirock, GC Corporation, Tokyo, Japan) and pressed lithium disilicate (IPS e.max Press, Ivoclar Vivadent, Schaan, Liechtenstein) ceramic veneers were fabricated. At the time of delivery, the tooth preparations were cleaned with pumice paste and chlorhexidine gluconate (Consepsis Scrub, Ultradent Products Inc, South Jordan, UT, USA), and complete isolation was provided with a rubber dam (Dental Dam, Nic Tone, Bucharest, Romania). The teeth were treated with hydrofluoric acid (Porcelain Etch, Ultradent Products Inc, South Jordan, UT, USA) for 20 s, rinsed, and air-dried. The restorations were treated with phosphoric acid (Total Etch, Ivoclar Vivadent, Schaan, Liechtenstein) for 60 s and rinsed and air-dried, and then silane (Monobond Plus, Ivoclar Vivadent, Schaan, Liechtenstein) was applied for 60 s and was followed by the application of a dental adhesive (OptiBond FL, Kerr, Orange, CA, USA). The teeth’s surface was treated with 20 micron aluminum oxide particles (AquaCare Aluminum Oxide Air Abrasion Powder, Velopex, London, UK), etched with 37% phosphoric acid (Total etch, Ivoclar Vivadent, Schaan, Liechtenstein) for 15 s, and conditioned with a primer and an adhesive (Optibond FL, Kerr, Orange, CA, USA), following the manufacturer’s recommendation. Finally, the ceramic veneers were cemented with dual-cure resin cement (Raviolini Esthetic, Ivoclar Vivadent, Schaan, Liechtenstein) (Figure 4), and each veneer was light cured for 20 s on each surface. After cementation, the patient was pleased with the contours, shade, and shape of the lithium disilicate veneers (Figure 5). Additionally, the patient received a clear polymethylmethacrylate (PMMA) occlusal guard to protect the restorations and was told to return to the clinic every 6 months to monitor the restorations. No complications were reported or noticed after cementation. The patient expressed satisfaction with the aesthetics of the ceramic restorations at the 1-year follow-up appointment (Figure 6).

## 3. Results of Treatment

The results obtained by the printed tooth reduction guide were optimum since the guide permitted uniform and controlled tooth preparation. The innovative design allowed the clinician to make vertical and horizontal reduction grooves easily; additionally, the clear resin permitted the continuous evaluation and visualization of the underlying tooth structure. This design with multiple channels is a feasible alternative to traditional silicone putty guides that only allow the visualization of a single area for tooth reduction. We also emphasize that total isolation with a rubber dam is required to ensure optimum bonding. Rubber dams provide several advantages, such as protection of the patient’s airway and the prevention of contamination with saliva. It is well-known that bonding on clean surfaces is necessary to obtain long-term success. At the end of the treatment, the patient was fully satisfied with the structurally durable and minimally invasive restorations and highly esthetic results achieved.

## 4. Discussion

CAD/CAM technology has revolutionized restorative dentistry. Digital dentistry has changed the majority of techniques for communication, the sharing and acquisition of information, and designing and producing tentative, provisional, and final prostheses for daily clinical work [31,32,33,34,35,36,37]. Three-dimensional printing permits the fabrication of diagnostic models [38] with higher accuracy and reproducibility than milled models [39]. Recent studies have proposed the replacement of traditional diagnostic casts with 3D printed models because additive technology has shown more precise results in terms of accuracy and reproducibility [29]. While our report and design are promising, it is a single case report and further validation in other clinical scenarios is necessary. A notable limitation of this case report is the lack of quantitative data comparing tooth reductions and preparation time for this guide to other 3D printed guides.

Complex appliances, such as tooth reduction guides, can be fabricated predictably with additive manufacturing technologies. Tooth reduction guides are indicated whenever a small portion of the tooth is tilted or rotated, or when there is mild tooth crowding or diastemas that can be adjusted prior to veneer preparations. However, if teeth are outside the arch or are extremely tilted or rotated, then a more conservation option, such as orthodontic treatment, can be employed prior to using tooth reduction guides [40,41,42]. Reports have described different designs for tooth reduction guides. Some allow reduction with hole access for very specific surfaces [43], some allow the preparation through traditional vertical access [44], and other types only provide a specific window for tooth reduction [45]. Unfortunately, the reported printed guides are very restrictive since they only allow preparing the tooth on a very specific surface and in only one direction. Our report describes the design of a reduction guide that permits controlled vertical, horizontal, and incisal tooth reduction. Therefore, this novel design functions as several reduction guides in one.

Tooth reduction guides have been shown to provide more accuracy for tooth preparation procedures. A recent in vitro study evaluated the amount of tooth reduction necessary for veneer preparations on twisted maxillary central incisors. The study compared the preparation by freehand of a thermoplastic guide, a silicone guide, a 3D printed uniform, and 3D printing with auto-stop features. The results indicated that both 3D printed guides, the uniform (0.093 mm), and the auto-stop (0.059 mm) features provided the least tooth removal, followed by the thermoplastic guide (0.149 mm) and the silicone guide (0.191 mm). Freehand preparation resulted in the most inaccurate and excessive tooth reduction provided, which was 0.237 mm [42]. Another study evaluated tooth reduction for veneer preparation on the cervical third, middle third, and incisal thirds of maxillary central incisors using freehand preparation, a silicone guide, a thermoplastic guide, 3D printing with a uniform guide, and 3D printing with auto-stop features. The results showed that freehand resulted in significantly more tooth reduction in all thirds prepared, while both types of 3D printed guides offered the least amount of tooth reduction and the most accurate preparation [27]. The two types of 3D guides can be classified as a 3D printed uniform and 3D printing with auto-stop. The uniform guide provides more freedom to the clinician to prepare the desired depth, and studies have shown that both types of guides provide similar accuracy and conservative tooth preparation than other types of guides. Future work will include evaluating our tooth reduction guide with others in terms of accuracy. Further additional work, starting in vitro, is necessary to quantitively demonstrate that this reduction guide design reduces clinical chair time and results in similar reductions as gold-standard guides. Such data could then be used to inform and obtain approval for a clinical trial for our prototype design.

Rubber dam isolation has long been recommended in restorative dentistry [46]. Even though the placement of the rubber dam has been criticized for being time-consuming [47], the advantages it offers are significant and justify its use. A study found that it takes an average of five minutes and four seconds for a student without experience to place a rubber dam [48]. It is important to mention that experience reduced this time significantly. Rubber dam isolation has been reported to protect the patient’s airway, reduce aerosol contamination, maintain a clean working area, and prevent bonding surface contamination with saliva [49,50,51,52,53]. A clinical in situ study evaluated the bond strength of thirty molars restored with direct resin composite restorations bonded using two dental adhesives with and without rubber dam isolation. The results showed that the restorations placed with rubber dam isolation had higher bond strengths than those placed without it [54]. In this report, the ceramic veneers were cemented with total rubber dam isolation to maximize the bonding between the different materials and the tooth structure.

Currently, clinicians have several ceramic options for dental veneers such as composite, feldspathic porcelain, leucite-reinforced glass ceramics, lithium disilicate, zirconia, and combinations among them [55,56,57]. Great long-term survival rates have been reported for veneers bonded to enamel; therefore, a conservative preparation is recommended to ensure long-term success [58,59]. A recent study evaluated the clinical performance of 1074 lithium disilicate veneers over a period of 4 years and concluded that when proper bonding protocols are used, these restorations had a survival rate of 99.83% [60]. A retrospective analysis evaluated the survival rate of 364 lithium disilicate veneers for a total of 10 years. These restorations displayed a success rate of 97.4% with almost no complications [61]. Due to the successful long-term record of this material, in our report, the authors decided to use lithium disilicate as the ceramic of choice to fabricate the veneer restorations.

## 5. Conclusions

In conclusion, the development of 3D printing technology has opened up new possibilities in restorative digital dentistry. With additive manufacturing techniques, dental clinicians can now create custom appliances on demand that facilitate precise, uniform, and conservative tooth preparation. The proposed novel 3D printed tooth reduction guide design features horizontal and vertical access, which is a significant improvement compared to traditional guides that only permit visualization of the tooth on limited surfaces. This guide enabled a predictable placement of the ceramic laminate veneers, with a one-year follow-up demonstrating satisfactory results. The guide’s innovative design provides clinicians with greater flexibility, precision, and control over tooth preparation, minimizing the risk of overpreparation while achieving optimal aesthetic outcomes. However, future work is needed to directly measure the accuracy of this new guide compared to other traditional methods. Overall, this case report underscores the potential of additive technology to revolutionize dental restoration, and the authors expect that these findings will encourage further exploration and development in this exciting field.

## Data Availability

Not applicable.
